# 2,2′-Dihydroxy-*N*,*N*′-(ethane-1,2-di­yl)di­benzamide

**DOI:** 10.1107/S1600536812051963

**Published:** 2013-01-09

**Authors:** Daniel Pereira da Costa, Sabrina Madruga Nobre, Bruna Gonçalves Lisboa, Juliano Rosa de Menezes Vicenti, Davi Fernando Back

**Affiliations:** a Escola de Quimica e Alimentos, Universidade Federal do Rio Grande, Av. Italia, km 08, Campus Carreiros, 96203-900 Rio Grande-RS, Brazil; b Departamento de Quimica, Universidade Federal de Santa Maria, Av. Roraima Campus, 97105-900 Santa Maria-RS, Brazil

## Abstract

The asymmetric unit of the title compound, C_16_H_16_N_2_O_4_, contains one half-mol­ecule, the whole mol­ecule being generated by an inversion center located at the mid-point of the C—C bond of the central ethane group. An intra­molecular O—H⋯O hydrogen bond forms an *S*(6) ring motif. In the crystal, mol­ecules are connected *via* N—H⋯O hydrogen bonds, generating infinite chains along [1-10].

## Related literature

For the synthesis of bis­amides, see: Apurba *et al.* (2002 ▶); Fry *et al.* (2010 ▶). For similar bis­amide crystal structures, see: Booysen *et al.* (2009 ▶). For applications of bis­amides as catalysts, see: Maurya *et al.* (2003 ▶); Liu *et al.* (2011 ▶).
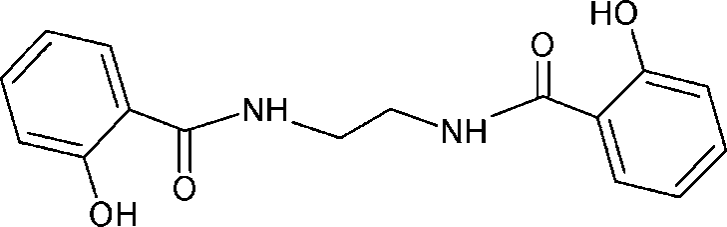


## Experimental

### 

#### Crystal data

C_16_H_16_N_2_O_4_
*M* *_r_* = 300.31
Triclinic, 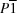

*a* = 4.6311 (3) Å
*b* = 5.0435 (3) Å
*c* = 15.2957 (9) Å
α = 89.091 (4)°
β = 83.315 (4)°
γ = 85.956 (4)°
*V* = 353.94 (4) Å^3^
*Z* = 1
Mo *K*α radiation
μ = 0.10 mm^−1^
*T* = 296 K
0.34 × 0.24 × 0.11 mm

#### Data collection

Bruker APEXII CCD diffractometer
Absorption correction: gaussian (*SADABS*; Bruker, 2009 ▶) *T*_min_ = 0.966, *T*_max_ = 0.989
9142 measured reflections
1535 independent reflections
924 reflections with *I* > 2σ(*I*)
*R*_int_ = 0.033

#### Refinement

*R*[*F* ^2^ > 2σ(*F* ^2^)] = 0.049
*wR*(*F* ^2^) = 0.143
*S* = 1.01
1535 reflections
100 parameters
H-atom parameters constrained
Δρ_max_ = 0.13 e Å^−3^
Δρ_min_ = −0.16 e Å^−3^

### 

Data collection: *APEX2* (Bruker, 2009 ▶); cell refinement: *SAINT* (Bruker, 2009 ▶); data reduction: *SAINT*; program(s) used to solve structure: *SHELXS97* (Sheldrick, 2008 ▶); program(s) used to refine structure: *SHELXL97* (Sheldrick, 2008 ▶); molecular graphics: *DIAMOND* (Brandenburg, 2006 ▶); software used to prepare material for publication: *publCIF* (Westrip, 2010 ▶).

## Supplementary Material

Crystal structure: contains datablock(s) I, global. DOI: 10.1107/S1600536812051963/lr2096sup1.cif

Structure factors: contains datablock(s) I. DOI: 10.1107/S1600536812051963/lr2096Isup2.hkl

Supplementary material file. DOI: 10.1107/S1600536812051963/lr2096Isup3.cml

Additional supplementary materials:  crystallographic information; 3D view; checkCIF report

## Figures and Tables

**Table 1:** Hydrogen-bond geometry (Å, °)

*D*—H⋯*A*	*D*—H	H⋯*A*	*D*⋯*A*	*D*—H⋯*A*
O1—H1⋯O2	0.82	1.76	2.495 (2)	148
N—H0⋯O1^i^	0.86	2.21	2.993 (2)	151

Symmetry code: (i) 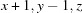.

**Fig. 1.**
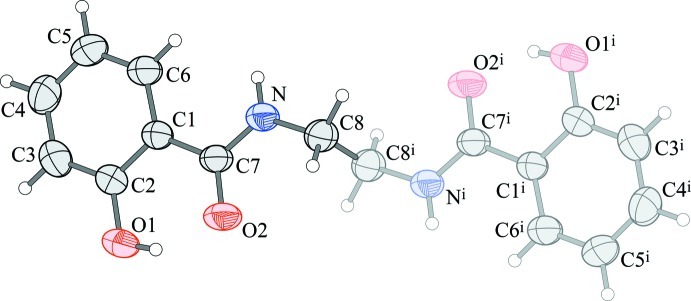
Molecular projection showing the asymmetric unit represented in dark colors. Ellipsoid probability: 50%. Symmetry codes: (i) 2–x, 1–y, 2–z

**Fig. 2.**
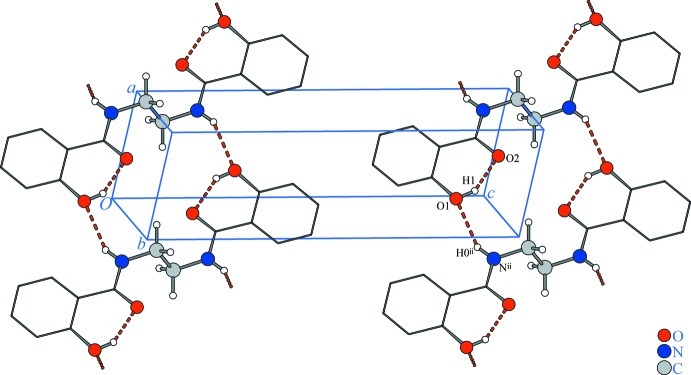
Packing diagram view showing the intramolecular e intermolecular hydrogen bond interactions. Some hydrogen atoms were omitted for clarity. (ii) -1 + x, 1 + y, z

## supplementary crystallographic information

### Comment

Nitrogenated compound amines, imines and amides are part of a broad class of molecules having a pharmacological and technological profile (Fry *et al.* 2010). Bisamides can be synthesized from a reaction between a diamine and an acylhalide under reflux (Apurba *et al.* 2002 ; Fry *et al.* 2010). Some synthesized bisamides have had their crystal structure determined (Booysen *et al.* 2009). However, others have been synthesized and applied to several reactions, such as the hydroformylation of phenol (Maurya *et al.* 2003) and due to the simplicity of the synthesis of these compounds, they have being applied to coupling reactions, such as the Suzuki reaction (Liu *et al.* 2011). The asymmetric unit is formed by one half of the molecule and the whole molecule is generated through a crystallographic center of symmetry placed in the mid-point of the C-C bond of the ethane bridging moiety (Fig. 1). Intramolecular and intermolecular classical hydrogen bond interactions are observed in the solid state. Each molecule participates in intermolecular hydrogen bond interactions with two other neighboring molecules through N^ii^–H0^ii^···O1 with a distance of 2.210 Å (symmetry code: (ii) -1 + *x*, 1 + *y*, *z*). The intramolecular hydrogen bonding systems forms six membered rings, showing a shorter distance of 1.763 Å (O2···H1–O1). These hydrogen bonding may contribute to the stabilization of the crystal structure (Fig. 2).

### Experimental

Ethylenediamine (0.1 mol, 6.6 ml) was added to a solution containing methyl salicylate (0.2 mol, 25.7 ml). The mixture was refluxed for 7 h. The reaction product was washed with ethyl acetate (3 times in 30 ml) and dried over CaCl_2_. After recrystallization from hexane, a yellow solid was obtained (23.1 g, 77%).

### Refinement

H atoms attached to aromatic C atoms were positioned with idealized geometry and were refined isotropic with *U*_eq_(H) set to 1.2 times of the *U*_eq_(C) using a riding model with C–H = 0.93 Å. H atoms attached to N atoms and to the ethylene fragment were located in difference Fourier maps. Their positional and isotropic displacement parameters were refined.

### Crystal data

**Table:**

C_16_H_16_N_2_O_4_	*Z* = 1
*M**_r_* = 300.31	*F*(000) = 158
Triclinic, *P*1	*D*_x_ = 1.409 Mg m^−^^3^
Hall symbol: -P 1	Mo *K*α radiation, λ = 0.71073 Å
*a* = 4.6311 (3) Å	Cell parameters from 1575 reflections
*b* = 5.0435 (3) Å	θ = 2.7–25.8°
*c* = 15.2957 (9) Å	µ = 0.10 mm^−^^1^
α = 89.091 (4)°	*T* = 296 K
β = 83.315 (4)°	Block, colourless
γ = 85.956 (4)°	0.34 × 0.24 × 0.11 mm
*V* = 353.94 (4) Å^3^	

### Data collection

**Table:**

Bruker APEXII CCD diffractometer	1535 independent reflections
Radiation source: fine-focus sealed tube	924 reflections with *I* > 2σ(*I*)
Graphite monochromator	*R*_int_ = 0.033
φ and ω scans	θ_max_ = 27.1°, θ_min_ = 2.7°
Absorption correction: gaussian (*SADABS*; Bruker, 2009)	*h* = −5→5
*T*_min_ = 0.966, *T*_max_ = 0.989	*k* = −6→6
9142 measured reflections	*l* = −19→19

### Refinement

**Table:**

Refinement on *F*^2^	Primary atom site location: structure-invariant direct methods
Least-squares matrix: full	Secondary atom site location: difference Fourier map
*R*[*F*^2^ > 2σ(*F*^2^)] = 0.049	Hydrogen site location: inferred from neighbouring sites
*wR*(*F*^2^) = 0.143	H-atom parameters constrained
*S* = 1.01	*w* = 1/[σ^2^(*F*_o_^2^) + (0.0644*P*)^2^ + 0.0596*P*] where *P* = (*F*_o_^2^ + 2*F*_c_^2^)/3
1535 reflections	(Δ/σ)_max_ < 0.001
100 parameters	Δρ_max_ = 0.13 e Å^−^^3^
0 restraints	Δρ_min_ = −0.16 e Å^−^^3^

### Special details

**Table:**

Geometry. All esds (except the esd in the dihedral angle between two l.s. planes) are estimated using the full covariance matrix. The cell esds are taken into account individually in the estimation of esds in distances, angles and torsion angles; correlations between esds in cell parameters are only used when they are defined by crystal symmetry. An approximate (isotropic) treatment of cell esds is used for estimating esds involving l.s. planes.
Refinement. Refinement of *F*^2^ against ALL reflections. The weighted *R*-factor *wR* and goodness of fit *S* are based on *F*^2^, conventional *R*-factors *R* are based on *F*, with *F* set to zero for negative *F*^2^. The threshold expression of *F*^2^ > σ(*F*^2^) is used only for calculating *R*-factors(gt) *etc*. and is not relevant to the choice of reflections for refinement. *R*-factors based on *F*^2^ are statistically about twice as large as those based on *F*, and *R*- factors based on ALL data will be even larger.

### Fractional atomic coordinates and isotropic or equivalent isotropic displacement parameters (Å^2^)

**Table:**

	*x*	*y*	*z*	*U*_iso_*/*U*_eq_	
O1	0.4041 (3)	1.1108 (3)	0.79344 (10)	0.0718 (5)	
H1	0.4677	1.0900	0.8411	0.108*	
O2	0.7318 (3)	0.9317 (3)	0.90207 (9)	0.0629 (5)	
N	1.0113 (3)	0.5534 (3)	0.87856 (10)	0.0507 (5)	
H0	1.0709	0.4248	0.8433	0.061*	
C1	0.7459 (4)	0.7361 (3)	0.76153 (11)	0.0426 (5)	
C2	0.5357 (4)	0.9263 (4)	0.73646 (12)	0.0496 (5)	
C3	0.4584 (5)	0.9234 (5)	0.65190 (15)	0.0676 (6)	
H3	0.3184	1.0495	0.6352	0.081*	
C4	0.5845 (5)	0.7384 (4)	0.59253 (14)	0.0649 (6)	
H4	0.5312	0.7405	0.5357	0.078*	
C5	0.7895 (5)	0.5493 (5)	0.61620 (14)	0.0655 (6)	
H5	0.8742	0.4220	0.5760	0.079*	
C6	0.8674 (5)	0.5507 (4)	0.69979 (13)	0.0589 (6)	
H6	1.0068	0.4226	0.7156	0.071*	
C7	0.8293 (4)	0.7466 (4)	0.85175 (12)	0.0448 (5)	
C8	1.1112 (4)	0.5513 (4)	0.96491 (12)	0.0523 (5)	
H8A	1.1517	0.7304	0.9796	0.063*	
H8B	1.2913	0.4402	0.9636	0.063*	

### Atomic displacement parameters (Å^2^)

**Table:**

	*U*^11^	*U*^22^	*U*^33^	*U*^12^	*U*^13^	*U*^23^
O1	0.0882 (11)	0.0606 (9)	0.0622 (9)	0.0348 (8)	−0.0120 (8)	−0.0122 (7)
O2	0.0780 (10)	0.0543 (9)	0.0545 (9)	0.0202 (7)	−0.0125 (7)	−0.0214 (7)
N	0.0591 (10)	0.0484 (10)	0.0439 (9)	0.0123 (8)	−0.0115 (7)	−0.0116 (7)
C1	0.0458 (10)	0.0388 (10)	0.0426 (10)	0.0022 (8)	−0.0050 (8)	−0.0043 (8)
C2	0.0569 (11)	0.0402 (10)	0.0501 (11)	0.0053 (9)	−0.0049 (9)	−0.0011 (9)
C3	0.0806 (15)	0.0620 (14)	0.0601 (13)	0.0147 (12)	−0.0198 (12)	0.0036 (11)
C4	0.0828 (16)	0.0679 (15)	0.0459 (12)	−0.0029 (12)	−0.0165 (11)	−0.0007 (11)
C5	0.0820 (15)	0.0642 (14)	0.0490 (12)	0.0113 (12)	−0.0094 (11)	−0.0158 (10)
C6	0.0696 (13)	0.0567 (13)	0.0488 (12)	0.0176 (10)	−0.0111 (10)	−0.0120 (10)
C7	0.0458 (10)	0.0420 (10)	0.0456 (10)	0.0040 (8)	−0.0041 (8)	−0.0085 (8)
C8	0.0540 (11)	0.0563 (12)	0.0477 (11)	0.0058 (9)	−0.0154 (8)	−0.0091 (9)

### Geometric parameters (Å, º)

**Table:**

O1—C2	1.349 (2)	C3—C4	1.365 (3)
O1—H1	0.8206	C3—H3	0.9300
O2—C7	1.245 (2)	C4—C5	1.372 (3)
N—C7	1.334 (2)	C4—H4	0.9300
N—C8	1.449 (2)	C5—C6	1.369 (3)
N—H0	0.8600	C5—H5	0.9300
C1—C6	1.383 (2)	C6—H6	0.9300
C1—C2	1.400 (3)	C8—C8^i^	1.511 (4)
C1—C7	1.478 (2)	C8—H8A	0.9700
C2—C3	1.382 (3)	C8—H8B	0.9700
			
C2—O1—H1	109.5	C6—C5—C4	119.07 (19)
C7—N—C8	122.58 (15)	C6—C5—H5	120.5
C7—N—H0	118.6	C4—C5—H5	120.5
C8—N—H0	118.8	C5—C6—C1	122.21 (18)
C6—C1—C2	117.95 (17)	C5—C6—H6	118.9
C6—C1—C7	123.62 (16)	C1—C6—H6	118.9
C2—C1—C7	118.42 (15)	O2—C7—N	120.36 (17)
O1—C2—C3	119.21 (17)	O2—C7—C1	120.87 (16)
O1—C2—C1	121.37 (17)	N—C7—C1	118.78 (15)
C3—C2—C1	119.42 (17)	N—C8—C8^i^	112.0 (2)
C4—C3—C2	120.98 (19)	N—C8—H8A	109.2
C4—C3—H3	119.5	C8^i^—C8—H8A	109.2
C2—C3—H3	119.5	N—C8—H8B	109.2
C3—C4—C5	120.4 (2)	C8^i^—C8—H8B	109.2
C3—C4—H4	119.8	H8A—C8—H8B	107.9
C5—C4—H4	119.8		
			
C6—C1—C2—O1	178.94 (18)	C2—C1—C6—C5	0.3 (3)
C7—C1—C2—O1	−2.0 (3)	C7—C1—C6—C5	−178.7 (2)
C6—C1—C2—C3	−0.3 (3)	C8—N—C7—O2	−1.8 (3)
C7—C1—C2—C3	178.77 (19)	C8—N—C7—C1	178.24 (17)
O1—C2—C3—C4	−179.4 (2)	C6—C1—C7—O2	173.61 (19)
C1—C2—C3—C4	−0.2 (4)	C2—C1—C7—O2	−5.4 (3)
C2—C3—C4—C5	0.6 (4)	C6—C1—C7—N	−6.5 (3)
C3—C4—C5—C6	−0.6 (4)	C2—C1—C7—N	174.55 (17)
C4—C5—C6—C1	0.2 (4)	C7—N—C8—C8^i^	80.7 (3)

Symmetry code: (i) −*x*+2, −*y*+1, −*z*+2.

### Hydrogen-bond geometry (Å, º)

**Table:**

*D*—H···*A*	*D*—H	H···*A*	*D*···*A*	*D*—H···*A*
O1—H1···O2	0.82	1.76	2.495 (2)	148
N—H0···O1^ii^	0.86	2.21	2.993 (2)	151

Symmetry code: (ii) *x*+1, *y*−1, *z*.
